# Early Integration of Palliative Care in Hospitals: How Can Palliative Care Consultation Teams Drive Practice Change?

**DOI:** 10.1177/23333936261421581

**Published:** 2026-02-20

**Authors:** Susanna Böling, My Engström, Johan Berlin, Joakim Öhlén

**Affiliations:** 1 Institute of Health and Care Sciences, Sahlgrenska Academy, University of Gothenburg, Sweden; 2 Palliative Centre, Sahlgrenska University Hospital, Gothenburg, Region Västra Götaland, Sweden; 3 Department of Surgery, Sahlgrenska University Hospital, Gothenburg, Region Västra Götaland, Sweden; 4 Department of Social and Behavioural Studies, University West, Trollhättan, Sweden; 5 School of Public Administration, Faculty of Social Science, University of Gothenburg, Sweden; 6 Centre for Person-Centred Care, University of Gothenburg, Sweden

**Keywords:** delivery of health care, referral and consultation, palliative care, palliative medicine, pancreatic neoplasms, qualitative research, quality improvement, interprofessional relations

## Abstract

Early integration of palliative care within disease-oriented care is advocated but the question of how this is best accomplished remains. In the context of surgical care for patients with pancreatic cancer, a quality improvement initiative was introduced whereby palliative care consultations were offered early in the disease trajectory. We devised a qualitative study using an interpretive description design, focusing on the integration of palliative care consultation practice and collaboration between actors. The aim of the study was to examine a practice-driven change for the early integration of palliative care within surgical cancer care. Seventeen study participants (healthcare professionals, managers and patient association representatives) were interviewed. The interviews were complemented by observations, and a constant comparative analysis was applied. This study found that the development and success of the quality improvement initiative were shaped by the interplay among three key actors – the palliative care team, the surgical team, and the patient and their family – and were further influenced by organisational factors, project structure and implementation, and broader societal circumstances. Within these domains, perspectives on *what*, *when* and *how* to integrate palliative care proved pivotal and need to be disclosed when initiating collaboration between palliative care consultation services and specialist hospital teams.

## Background

Early integration of palliative care with oncological care has been argued, based on growing evidence of its benefits for the wellbeing of patients with cancer (Kaasa et al., 2018). Palliative care is an interprofessional approach to the care of patients and families, focusing on amelioration of suffering and improving quality of life, and can be given in parallel with disease-targeted treatment (Radbruch et al., 2020; World Health Organization, 2002). Compared to other medical specialities, palliative care is depicted as having a different care culture (Friedrichsen et al., 2021; Kaasa et al., 2018) representing a separate paradigm of focusing on the *person*. In contrast, oncology, for example, is said to focus on the *disease* (Kaasa et al., 2018). A commonly identified problem is the clash between the curative and a palliative treatment intent (Sarradon-Eck et al., 2019), whereby abandoning the curative approach may be seen as a failure (McDarby & Carpenter, 2019). This tension motivates studying the intersection between palliative care and other disciplines, since the success of integration requires joint efforts and collaboration between actors representing different perspectives on approaches to care.

Specialised palliative care is needed when patients with progressive, life-threatening illness experience complex problems, such as severe symptoms or other difficult situations like ethical challenges related to discontinuation of futile treatments. Moreover, patients who do not require specialised palliative care services are to be provided with non-specialist palliative care across all healthcare services (Radbruch et al., 2020). However, theories of public health palliative care emphasise the importance of integration between specialised and non-specialised palliative care services, civil society and informal support networks surrounding patients to ensure equitable access to quality palliative care for the population (Abel et al., 2018), underscoring the necessity of a holistic and proactive approach to early palliative care integration. To facilitate this, palliative care consultation services serve as a bridge between specialised and non-specialised palliative care. These services comprise specialised palliative care professionals who support other healthcare providers through, for example, consultation and education (Payne et al., 2022). Such an opportunity for consultation is seen as one step on the path to achieving national palliative care integration (Radbruch et al., 2020). In regard to integration of oncology and palliative care, Kaasa et al. (2018, p. 5) have stated that ‘integration aims to coordinate the activities of professionals, with the overall goal of improving patient care’, which ‘requires change at the system level on the basis of a common understanding and acceptance of the two paradigms’.

Previous studies have shown that palliative care consultation services have positive effects on end-of-life quality (Chen et al., 2024), symptom control, care planning and reduction of futile treatment (Janberidze et al., 2021). Particularly when these services are integrated early on, there is evidence of potential improvements in patient care satisfaction and a reduction in hospital length of stay (Helgeson et al., 2023). Nevertheless, palliative care consultation practices may vary (Böling et al., 2020), prompting an investigation of what works in practice and for which patient groups.

This study focuses on a quality improvement initiative for patients with pancreatic cancer, which is a lethal disease, with a 5-year survival rate of 6.6% (Regionala cancercentrum i samverkan [Regional Cancer Centres in Sweden], 2022). Patients in this group often have considerable care needs due to symptoms like pain (Lakatos et al., 2016; Tang et al., 2018), decreased appetite, fatigue (Tang et al., 2018) and gut problems (Gooden & White, 2013). In a cohort of 5,381 patients with advanced pancreatic cancer, patients survived 147 days on average, with 50% of the cohort dying within 75 days (Jang et al., 2015). Studying this patient group from a palliative care integration perspective is of great interest due to the specific disease trajectory of its patients, who often initially experience good health with comparatively little impact on their wellbeing followed by rapid deterioration, requiring healthcare professionals and care systems to act proactively and swiftly.

Despite the documented positive effects of palliative care on wellbeing (Maltoni et al., 2016), aggressive end-of-life care (Jang et al., 2015), care planning, and lack of patient information (Walling et al., 2016), Karlekar et al. (2014) present challenges to palliative care referral in the surgical context. Obstacles to implementing palliative care have been identified among surgeons on a systemic, clinician and patient level (Suwanabol et al., 2018). Moreover, the disease trajectories of patients with surgical illnesses have been considered different to those of patients with other chronic illnesses, due to, for example, temporary post-operative symptoms and high risk of sudden deterioration. Further research is thus merited (Lilley et al., 2018) in regard to models of palliative care delivery (Lilley et al., 2016; Walling et al., 2016) and implementation strategies for increased basic palliative knowledge within surgical care (Lilley et al., 2018). Earlier research has identified factors that influence collaboration between hospital teams and palliative care consultation teams, such as effective communication, unclear role definitions, motivation through skill-building, and the importance of being visible and responsive (Firn et al., 2016). However, the majority of these studies have been conducted in the United States and the United Kingdom, where in-hospital palliative care consultations are often common practice, highlighting the need for research in other healthcare systems (Firn et al., 2016).

Our motivation for this study stems from a long-term interest in contributing to how palliative consultation services can facilitate early integration of palliative care. The endeavour is to further our understanding of factors that influence early integration of palliative care within practice-driven palliative care integration initiatives and increase our understanding of the interplay between palliative care consultation services and patient-responsible care clinics. The aim of the study was to examine a practice-driven change intended to promote early integration of palliative care within surgical cancer care for patients with pancreatic cancer. The research questions applied were: What are healthcare professionals’ perspectives on the integration of a palliative care consultation practice for early integrated palliative care within surgical care? Which factors seem to influence the collaboration processes, integration and impact of the quality improvement initiative?

## The Quality Improvement Initiative

**Figure 1.**
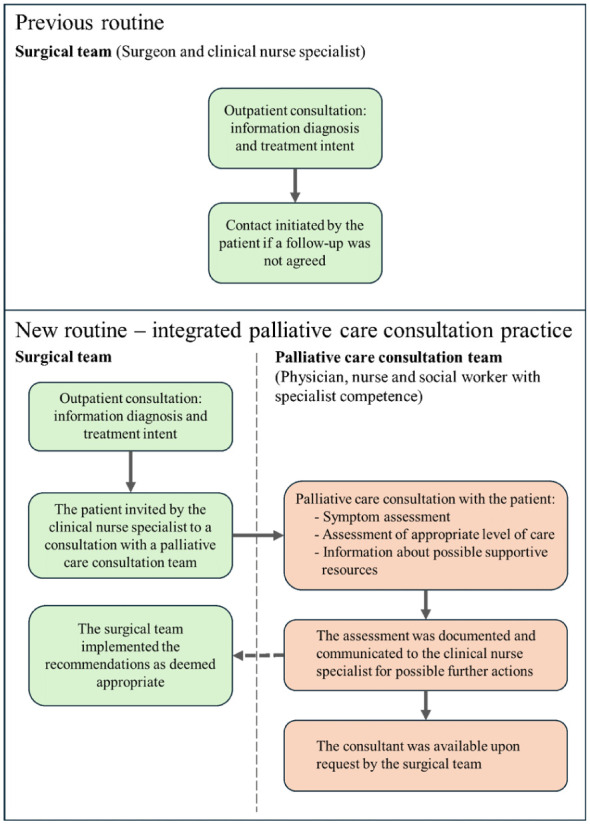
Previous routine and new, integrated palliative care consultation practice. Adapted with permission from Böling (2025).

A university hospital surgical clinic and a palliative care consultation team initiated a collaborative practice-led quality improvement initiative with the aim of developing and testing a model for early integration of palliative care for patients with non-curable pancreatic cancer. The project development, which is further described in Supplemental File 1, took a cyclical approach by developing, testing and evaluating the model (Coghlan & Brannick, 2014). After initial discussions and planning, the two teams introduced the quality improvement practice (Figure 1) whereby palliative care consultations were offered early (close to diagnosis) to patients with non-curable pancreatic cancer in addition to standard care (Figure 1, Supplemental File 1). During the patient diagnosis and treatment consultation, the surgical clinical nurse specialist offered the patient a meeting with a palliative care consultation team. For patients who accepted this offer, the palliative care consultation team met with the patient and their family, assessed their palliative care needs and provided information and guidance. The palliative team’s recommendations were communicated to the clinical nurse specialist within the surgical team, after which the surgical team decided which recommendations to implement and remained responsible for the patient’s ongoing care. The palliative care consultation team was available for further consultation to the surgical team, if needed. The quality improvement practice design is described further in Supplemental File 1. The project was initiated in 2019, paused for 6 months during the Covid-19 pandemic and closed in 2022. Approximately 14 patients were seen by the palliative care consultation team during the project.

The quality improvement initiative stemmed from a long-standing clinical collaboration with a shared interest in integrating palliative care principles into surgical care. The lead researcher was external to both teams, while two team members held joint positions bridging academia and the respective clinical organisations. The research team observed the quality improvement initiative throughout its course but did not participate in patient care or in the design of the quality improvement practice. The research team’s diverse backgrounds in nursing, palliative and surgical care, and organisational studies provided both insider and outsider perspectives, which were continuously reflected upon to enhance reflexivity.

## Method

### Design

The study adopted an overall Interpretive description design, which emphasises creating knowledge driven by practice knowledge interests (Thorne, 2016). The study was ethically approved by the Swedish Ethical Review Authority (No. 2022-02272-02) and based on an initial application to the Regional Ethical Review Board in Gothenburg (No. 809-16).

### Setting/Context

Sweden has a Beveridge tax-based, publicly funded healthcare system, which is highly decentralised. As a result, local governments have a high degree of independence in how they provide healthcare regionally. Consequently, palliative care consultations are practice driven (Böling et al., 2020), and national policies regarding their organisation and use remain vague (Socialstyrelsen [National Board of Health and Welfare], 2013). Sweden has initiated ongoing national healthcare reforms with the aim of achieving person-centred integrated care, and hospitals are still the most common places to die (Öhlén et al., 2025). According to Swedish policy, palliative care is recommended to be integrated throughout the healthcare system, and provided at both specialist and generalist levels (Socialstyrelsen [National Board of Health and Welfare], 2013). However, availability and organisation of palliative care vary across the country due to the decentralised governance of healthcare (Cancerfonden [The Swedish Cancer Society], 2024) and differences in how palliative care is incorporated into healthcare education curricula (Hagelin et al., 2022).

This study was conducted at a university hospital that includes a comprehensive cancer centre. Within this setting, the surgical clinic, here addressed as the patient-responsible care clinic, is the clinic holding overall medical responsibility for the patients. During the quality improvement initiative, palliative care consultation teams were available for consultation across all hospital clinics and also conducted regular rounds with some units. The specialised palliative care in-patient unit was temporarily closed. Organisation of specialised palliative home care services varied within the region. In some areas, such services were available 24/7, while in others they were available Monday to Friday during daytime hours. However, during the course of the quality-improvement initiative, the organisation of such services changed, and all regions had specialised palliative home care available during weekdays only. The Covid-pandemic started during the introduction of the quality-improvement practice and was ongoing throughout most of the project.

### Participants, Sampling and Data Generation

Different participant categories and qualitative data generation methods were included to enhance understanding of the integration of palliative care within the quality improvement initiative, as well as credibility of the findings (Thorne, 2016). Data generation was conducted concurrently with the quality improvement, enabling the researchers to examine the process in real time through observations of follow-up meetings between the teams. Additional informal conversations with team members also added to the understanding of the project. Following the completion of the quality improvement initiative, a purposive sample (Polit & Beck, 2021) of healthcare professionals and managers from the surgical and palliative care team were invited to a focus group and interviews. The researchers chose between interviews and focus groups based on logistical factors, such as the feasibility of assembling teams simultaneously, as well as considerations related to minimising potential hierarchical dynamics within the groups. Interviewees (Tables 1 and 2) were included if they had been involved in the development and/or integration of the quality improvement practice. Of the 21 invited participants, six declined the invitation or did not respond. In addition, participatory observations (Thorne, 2016) were conducted during the three follow-up meetings between the surgical and palliative team. This allowed the authors to pose follow-up questions and to some extent engage in the discussion, particularly when questions related to the research components arose. Moreover, two patient association representatives were included and interviewed to add patient and contextual perspectives, although these had not been involved in or affected by the quality improvement. Participants were invited via email and gave informed consent prior to the observations and interviews. The researchers aimed to maintain transparency and uphold participants’ voluntary participation, for instance, by reiterating the purpose of the study and seeking consent before initiating the audio recordings.

**Table 1.** Type of Data and Participants.

Type of data	Number of occasions	Participants
Observations	3 (26–59 min)	5 registered nurses (S, PCC), 1 social worker (PCC), 2 physicians (S, PCC), 1 manager^1^ (S)
Focus group	1 (1 hr 36 min)	2 registered nurses, 1 social worker, 1 physician (PCC only)
Interviews	10 (28 min–2 hr 39 min)	4 registered nurses (S, PCC), 1 physician (S), 2 patient association representatives, 4 managers^2^ (S, PCC)
Total (*n*)	14 (11 hr 24 min)	17 (7 registered nurses, 2 social workers, 2 physicians, 2 patient association representatives, 4 managers^2^)

*Note.* S = surgical team; PCC = palliative care consultation team.

1 Manager and registered nurse.

2 Of which three are registered nurses and one is a physician.

**Table 2.** Participant Characteristics.

Participant characteristics		years/number of participants
Age		
	Mean	50 years
	Range	33–78 years
Sex		
	Female	12
	Male	5
*Profession* ^a^		
Surgical clinic	Nurse	6^b^
	Physician	2^c^
Consultation service	Nurse	4^c^
	Physician	1
	Social worker	2
*Professional specialisation* ^a^		
Surgical clinic	Surgical care specialist nurse	2
	Other nurse specialist	1
	Surgical consultant	2
Consultation service	Palliative care specialist nurse	2
	Other nurse specialist	2
	Specialist consultant other than palliative care	1
*Healthcare professionals’ experience in their current practice*		
Surgical clinic	Mean	8 years
	Median	5 years
	Range	2–20 years
Consultation service	Mean	2 years
	Median	1 years
	Range	0–6 years
*Palliative care practitioners’ experience of palliative care*		
	Mean	10 years
	Median	8 years
	Range	1–30 years

*Note*. ^a^Patient representatives excluded.

b Including two managers.

c Including one manager.

The focus group (Barbour, 2018) and interviews with healthcare professionals and managers were semi-structured, adapted to the interviewee’s role and based on three to five main questions with possible follow-up questions (Supplemental File 2). Some of the questions were influenced by Grol et al.’s (2013) framework of determinants for implementation, namely characteristics of the ‘innovation’ (here viewed as the quality improvement practice), ‘users’, ‘target group’, ‘social environment’, ‘context’, and ‘efficiency of the implementation strategies’. During the observations, the authors focused on the collaboration and development of the integrated practice. Observations, interviews and the focus group were audio recorded, and interviews with healthcare professionals and managers were transcribed verbatim. In accordance with the chosen study design (Thorne, 2016), the analysis started during the observations and interviews. Based on what was learnt during this process, interview questions were formulated, modified and added. The first author conducted the focus group and all but one of the interviews; the remaining interview was conducted by a co-author, who also co-facilitated the focus group. Both researchers had extensive experience in qualitative interviewing. The first author took analytical notes after each interview, including reflections on personal preconceptions and underlying theoretical assumptions that could influence interpretation (Thorne, 2016).

### Data Analysis

Interpretive description encourages the researcher to have a critical and interpretive mind in order to find ‘below surface meaning’ (Thorne, 2016, p. 192). The data analysis was an iterative process, which involved keeping an analytical diary throughout data generation and analysis (Thorne, 2016). The first author read the initial analytical notes and listened to the audio recordings while performing a comprehensive reading of the transcripts. After the comprehensive reading, an analytical understanding of the material was formed. From the emerging analysis, broad categories were formulated under which the interviews and observations were coded in the software NVivo, version 1.7. To facilitate the process of identifying patterns and connections within data, data were coded under multiple categories when relevant. A few additional categories were added during the coding process. After all transcripts were coded, each category was read and summarised. This was followed by a process in which the first author tried out different ways of organising and understanding the whole and its parts. An analytical result was formulated, starting with the concepts closer to the text, and continuing with the overarching ones (Thorne, 2016). Constant comparative analysis was applied throughout the analysis process, comparing parts with parts and parts with the whole to find patterns within the data (Charmaz, 2014; Thorne, 2016). Subsequently, there was a follow-up meeting with the healthcare professionals from the surgical and palliative care teams to provide the participants with feedback on the preliminary findings. This was followed by a short discussion, which could be seen as a way of fuelling continued analytical thinking, thereby enhancing credibility (Thorne, 2016). As the analysis was coming to an end, the first author listened to the interviews with patient representatives, whose reasoning was primarily used in the analysis to provide contextual insight and to enhance understanding of patients’ situations and needs. Interviews with managers added perspectives on the surgical and palliative care context, but also experiences of the quality improvement initiative itself.

To increase credibility and trustworthiness of the findings, and to critically reflect on their preunderstandings, the authors maintained a continuous dialogue throughout the analysis process. The analysis and manuscript were also discussed in seminars with other researchers and practitioners.

## Results

**Figure 2.**
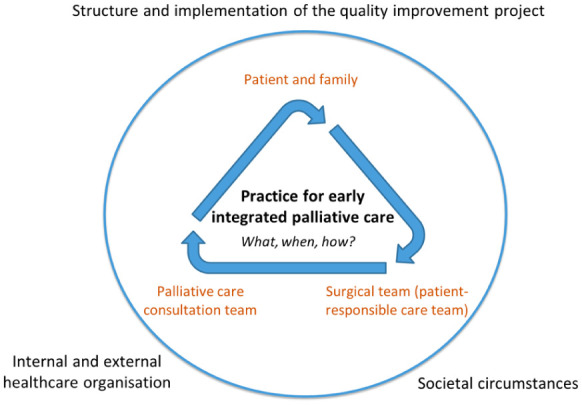
Collaborative development of a practice for integrated palliative care as a dynamic interplay at the intersection of the surgical team (patient-responsible care team), the palliative care consultation team, and the patient and their family.

We found that the development and success of the quality improvement project were shaped at the intersection of three main actors – the palliative care team, the surgical team, and the patient and their family – and influenced by both internal and external healthcare organisational factors, the project’s structure and implementation, as well as the broader societal circumstances (Figure 2). The collaboration constituted a process that illuminated similarities and differences in perspectives, practices and needs. In different ways, the three actors interacted with and affected the quality improvement practice and its integration. In particular, divergent perspectives in relation to palliative care integration regarding *what* should be integrated, *when* and *how* seemed influential on the quality improvement project. The two care teams had diverging perspectives on several different aspects, such as the concept of palliative care, patient needs, integration and roles. The success of the quality improvement initiative was also limited by organisational differences in palliative care practices between the two teams. Patients were not involved in developing the practice design, but their condition and individual situations were pivotal to the success of the quality improvement initiative and highlighted key considerations for future quality improvement projects.

The results will present our analysis of the dynamic interplay at the intersection between the surgical team, the palliative care consultation team, and the patient and their family, and of how this interplay relates to and influences the quality improvement project. In this context, *interplay* refers not only to the collaboration between the actors, but also to how their perceptions, professional roles and responsibilities, and organisational circumstances affect their collaboration and the development of early palliative care within the quality improvement project. The analysis captures how the distinct contexts and preconditions of the surgical team, the palliative care team, and the patient–family unit shape, enable and constrain the collaborative practices that emerge within the quality improvement project. A more detailed overview of specific factors influencing the processes, collaboration and impact of the quality improvement project is provided in Supplemental File 3.

### Agreement and Divergence in Perspectives on Palliative Care and Patient Needs

In the interplay between the surgical nurses and the patient and family (Figure 2), perceptions and preconceptions of palliative care played an important role in the project’s feasibility, particularly regarding the process of offering patients a palliative care consultation. Nurses in the surgical clinic mentioned that, due to their own personal feelings and perceptions of palliative care, as well as their assumptions about patients’ views, they found it challenging to broach the subject with patients, and this seemed to be a key barrier to the practice. Palliative care was commonly considered a ‘sensitive topic’ and could therefore be hard to bring up, thus influencing whether or not the nurse chose to offer patients (particularly the younger ones) a palliative care consultation. Thus, the perspective of *what* palliative care entails was crucial for the project. This occurred despite the nurses’ increased awareness of palliative care not being restricted to end-of-life care, and despite their perceiving that the patient had received clear information regarding disease severity and had been informed that the treatment plan was limited to ‘best supportive care’. The clinical nurse specialists’ view was shared by the patient-association representatives, who believed that patients commonly viewed palliative care as equal to end-of-life care. A nurse shared her feelings about offering a palliative care consultation to a vital patient:

So [nurse 10] and I talked about it last week – that a lot rests with us personally too – our own perception of what it’s like to inform a patient about something like this when the patient is reasonably well. . . or palliative stuff like [nurse 10] mentioned – that’s pretty loaded. And it’s not always appropriate [and] if I still think it feels uncomfortable to talk about it on a first visit, when the patient looks really chirpy, then I’m reluctant to do so. And for the sake of the patient too – so they don’t feel like it’s all over. (Nurse 04, surgical clinic)

Furthermore, in relation to both teams’ perceptions of *what* palliative care entails, the consultation team highlighted that friction could arise due to differing perspectives on the patient’s needs and personal responsibilities in relation to their care. For example, the surgical ‘best supportive care’ practice meant that mainly the patients themselves were responsible for contacting the clinical nurse specialist for support during telephone hours. The consultation team felt this surgical practice was not proactive and conflicted with their view of palliative care and this patient group’s needs. The patient representatives also highlighted the importance of the responsible healthcare team being proactive in terms of initiating continuous contact with patients. One palliative care physician emphasised this perceived divergence of views and practices thus:

No, because we have such different mentalities about this and such different views about what these patients need. And I even asked one of them. . .yes, it must have been at the last meeting, ‘what does best supportive care mean to you?’ For me, best supportive care [actually] consists of something [. . .] and so you call it best supportive care. I don’t think you can call it anything. And that’s where it gets difficult for me. (Physician 01, consultant)

From another angle, one clinical nurse specialist shared the view that at times, the two teams had divergent perceptions of *what* was understood to constitute the patient’s needs. The perception was that the consultation team desired speedier plans and action than the surgical team, which could result in conflicting care planning. The clinical nurse specialist also commented on differing views of the ‘whole’ and considered palliative care to be narrower in scope than surgical care. For example, the surgical team included a dietician (who played a crucial role in caring for this specific patient group) while the palliative care team lacked a similarly prominent nutritional perspective. The nurse’s statement may reflect an underlying perception of palliative care as not being concerned with disease-specific supportive interventions. While limited integration between the two teams and their surgical and palliative care expertise was evident, communication improved and they became more in tune with each other over time. As the clinical nurse specialist said, ‘*I still feel we eventually found a fairly good way of working [together] despite the pandemic*’ (Nurse 04, surgical clinic).

In relation to palliative care, a surgeon and a nurse in the surgical team shared the view of palliative care team members that there was a divergence of care cultures. They described surgical care as having a dominant focus on the organs and specific problems, in contrast with palliative care, which required zooming out to apply a broader perspective. They shared this perspective with one of the surgical managers, who also stated that surgeons’ focus and intense interest in the ‘surgical’ impeded their palliative care knowledge. According to the palliative consultation team, these divergent perspectives on patient care and focus on the surgical prevented surgeons from adopting a palliative care approach and identifying palliative care needs. The interviewed surgeons had differing perceptions as to their profession’s palliative care competence, spanning from a perceived need for support to a feeling of already having extensive experience and thereby sound palliative care skills among colleagues. Such a perception of self-sufficiency in palliative care competence may either reflect an already sufficient level of palliative care practice or a lack of recognition of their knowledge gaps, meaning surgeons possibly miss out on consultation opportunities. This observation connects to broader questions about the perceived role and expected contributions of palliative care consultation services.

### Divergent Perspectives on Palliative Care Integration and the Consultant Role

Regarding the interplay between the two teams (Figure 2), the palliative care consultation team initially had the ambition of early integration of palliative care for patients with pancreatic cancer. However, the surgical team suggested they exclude patients planned for surgery (so called ‘curative treatment’) even though these patients were likely to be near end of life, indicating there were divergent perceptions of *when* integration of palliative care should occur. In the end, it was decided that patients who were neither considered for surgical treatment nor chemotherapy could be included. Later, there were discussions in follow-up meetings about broadening the patient group to include patients who had been referred for oncological treatment. However, the surgical team appeared somewhat reluctant to accept this suggestion due to an assumption that these patients were still holding on to hope. Upon further discussion, the palliative care physician agreed with the view that these patients should be excluded, stating that the involvement of another team might be overwhelming for the patients. Moreover, the physician noted that those undergoing chemotherapy were already closely linked to the oncological team, and therefore considered their palliative care needs to be adequately met within that setting.

The interviews highlighted differences in perspectives on the integration of palliative care within the quality improvement initiative, including what role the palliative consultation team should have in this, which relates to *how* to integrate palliative care. During the project, the palliative team had envisaged having a consultative role without patient responsibility and communicated this to the surgical team. However, once the project was up and running, some of the surgical team wanted the consultation service to take greater patient responsibility and implement more of their recommendations themselves. As one clinical nurse specialist stated: ‘*some doctors, or lots of them, think that because the consultation team have met the patient face to face, they might as well have done that too, really to save time*’ (Nurse 04, surgical clinic). This was grounded in the view that it would result in more efficient care processes, reduced burden for surgeons and avoid delay in implementing recommendations. It also followed a logic that referrals to specialised palliative care would take place within the same organisation as the consultation team. In the view of one surgeon, handover of patients could result in a more successful project, due to greater returns for both patients and physicians, as expressed in the quote below:

And I think it might have turned out better if they had taken on those patients. Actually, that would have been pretty logical, I think – than [. . .] ‘you’re asking me to write a referral to you’ – that’s a bit weird. I think it would have been better [if they had taken on those patients]. And then maybe it would have had a greater impact, possibly. Then it would have been easier for all our colleagues to think about it too, if you knew that this was something that benefited both the patient and me, actually, because I’m saving a bit of time, so to speak. (Physician 02, surgical clinic)

However, the palliative consultation team argued that, due to their intermediatory function, their role within the quality improvement practice was too fragmented and suboptimal, which underscores complexities in *how* palliative care can be integrated. They preferred more integrated practices, and gave examples of consultation practices with other departments, where they sat down and discussed patients and palliative care needs with the patient-responsible care team. They also perceived such collaborations to function better when their team actually met with the other team, as explained by this palliative care nurse:

But if we had been invited to the surgical clinic. . . like when we go to the ‘xx’ clinic now, for example. Then I think it would work. There, they flag patients they want to discuss with us – whatever is problematic. Because then, everyone is interested in getting help or working on something further or. . .it’s not as strained. (Nurse 06, consultation team)

### Differences in Organisation of Palliative Care Team Practice

Differences in *how* the two teams organised their palliative care practices influenced both their collaboration and the overall quality improvement project. Within the interplay between the two teams there was a mismatch in role distribution for palliative care – particularly around which profession led the palliative care practice. While the palliative team emphasised a team approach to patient care, the palliative medicine consultant served as the informal leader because medical recommendations were pivotal. Surgical palliative care practice was largely led by the nursing profession, with surgeons or other professions consulted when needed. This led to the palliative care physician noting a lack of contact with surgeons. It also meant that the palliative care team, in their consultancy role, had to communicate treatment and care recommendations to the clinical nurse specialist, rather than surgeons. When this consultative approach met surgical palliative care practice, it resulted not only in an increased workload for the clinical nurse specialists, but also in a risk of delaying patient measures, if the surgeon could not take action on the recommendations in a reasonable amount of time. These dynamics reflect differences in both *how* palliative care was operationalised through team composition, leadership and communication pathways, and *what* it was understood to entail in terms of responsibilities and core professional contributions.

### Unmet Patient Needs Within the Consultation Practice and the Surrounding Organisation of Specialised Palliative Care

In the interplay with the patient-family unit (Figure 2), the consultation practice was perceived as meeting patient needs and being something positive for patients and families, as it could provide information and support in care planning. The teams also perceived that family carers experienced greater contentment and a sense of security from being able to access consultants, who facilitated complementary holistic thinking, and more timely initiation of appropriate interventions. Nevertheless, both the palliative consultation team and the surgical team perceived the intersect between the patient and the two care teams and their respective roles to have undesirable effects. For example, the addition of a second care team could constitute a burden for the patient and cause confusion regarding care responsibility in terms of who the patient should turn to with different issues.

The success and appropriateness of the quality improvement practice were affected by the character of the patients’ condition, often with rapid deterioration, which delimited the time for approaching patients before they were in a state where they needed full-time specialised palliative care or died in the surgical ward. Thus, the relevance of the quality improvement practice for this particular patient group may be questioned, both regarding its timing (*when*) and its content (*what*). The palliative consultation team considered it beneficial to offer a selective measure of palliative care consultation to patients in a stable phase, without acute problems. However, for patients with considerable ongoing needs, immediate action was required, for which the practice was not adapted.

And I don’t know what you guys think, but it was just that once we were in that phase and could inform them of what was to come, that it wasn’t acute, then it often went quite well. But when it was like, ‘oh, now we’re here and we need help right now’ – like when the pain is impossible or it’s a crisis and panic now, and the patient can’t stay at home – that’s when it’s incredibly difficult to say ‘yes, but we’re here now just to give you a little advice’ or ‘we’re just going to tell you what you can do as next steps. (Social worker 03, consultant)

Right from the beginning of the collaboration, the surgical team highlighted the problem of having a patient group that would rapidly deteriorate and often end up in hospital, possibly dying there. They also highlighted a need for pending referrals to specialised palliative care, to enable rapid enrolment of the patient when needed. This was not possible in the current specialised palliative care organisation, which lacked a specialised palliative inpatient unit and was undergoing organisational changes. The surgical team further expressed a wish for the palliative consultants to refer or enrol patients to their own organisation, which, according to the consultant physician, was not possible due to organisational structures and the need for a referral from the patient-responsible care team.

The surrounding organisation of palliative care had an impact on *how* and *when* the consultation practice was considered relevant (Figure 2). One clinical nurse specialist emphasised the importance of considering the consultation role in relation to other specialised palliative care opportunities. For example, it could be more relevant to refer the patient to a specialised palliative home care or hospice than to offer a palliative care consultation. Although there was consensus between the palliative care team and the surgical team that healthcare (including palliative care) did not currently respond sufficiently to the specific conditions and needs of the patient group, the project consultation practice was not perceived to compensate for and fill the existing vacuum in the care chain. Rather, the consultation team argued they became one care provider too many (for a fragile patient group) and the consultant physician suggested finding alternative practices to support the palliative competence and practice of the clinical nurse specialists as a better solution, thereby strengthening the generalist palliative care provision.

### Contextual Influence of Societal Events, the Quality Improvement Project’s Structure and Implementation

In relation to the surrounding context and societal events (Figure 2), the Covid-19 pandemic, which started during the launch of the quality improvement practice, was perceived by the interviewees as one of the major obstacles towards its integration. Apart from putting the quality improvement on hold, the pandemic also presented an exceptional situation marked by shifting priorities, a constant flow of information, new routines and a physical distance between teams. Moreover, the limited number of patients available to be approached regarding a meeting with the palliative consultation team was also regarded as a considerable barrier to the project’s overall success.

The quality improvement change was also influenced in different ways by factors related to the structure and implementation of the quality improvement project (Figure 2), such as lack of knowledge, information and communication about the quality improvement. While this was partly attributable to the Covid-19 pandemic, it also likely stemmed from insufficient facilitative structures and the lack of influential internal advocates. In addition, there was a perceived lack of clarity and structure within the quality improvement project, which may have resulted from the high degree of flexibility in how it could be shaped and modified over time, in line with its collaborative, cyclical approach. In contrast, clear information at the start of the quality improvement, the establishment of routines, communication channels, motivation and perceiving the quality improvement practice as beneficial, were seen as enablers for the quality improvement change.

The surgical team were positive about the palliative care consultation team and stated that several aspects of the consultation practice were a good fit with their practice. They saw the project as a way of enhancing quality of care for these patients. The clinical nurse specialists and one surgeon perceived the collaboration as relationship-building and as reducing the threshold for contact in other matters. Another surgeon expressed appreciation for what they had learnt from collaborating with the palliative care consultation service prior to the quality improvement initiative, which was perceived to strengthen the general palliative care provision. Moreover, internal and external facilitators were seen as enablers, when these were present.

The palliative care consultation team also mentioned enabling factors related to the patient consultation process, like digital consultation tools, familiarity within the consultation team and establishing a consultation outline (Supplemental File 1).

## Discussion

The main finding was that the development and success of the quality improvement project were shaped in the interplay at the intersection of the palliative care consultation team, the surgical team and the patient and their family. It was also affected by the internal and external healthcare organisation, societal circumstances and the structure and implementation of the project. Furthermore, the way in which healthcare professionals perceived the concepts of palliative care, integration and roles, together with differences in palliative care practices and organisation, influenced the integration of the quality improvement project and its practice. Our findings underscore the need to clarify perspectives on *what*, *when*, and *how* (Kaasa et al., 2018) when initiating a quality improvement effort to integrate palliative care early. Moreover, the result confirms the known clash between a curative and a palliative treatment culture, such as differences and challenges in focusing on both the person and the disease, and that the success of integration requires joint efforts and collaboration between healthcare professionals (Kaasa et al., 2018). The palliative care consultations (as practised within this study) had limitations in relation to patient needs and could not compensate for gaps in the surrounding organisation of palliative care, thus influencing the perceived success of the quality improvement practice. One of the study’s important contributions to the global evidence base is the real-world knowledge that was gained by studying a bottom-up, practice-driven change as it unfolded, and from the perspectives and experiences of those who implemented the change.

Much of the current study’s focus has been on barriers to integration of the palliative care consultation practice for patients with pancreatic cancer. From a different perspective we could argue that despite the immense challenges (e.g., during the Covid pandemic) patients were to some extent offered the opportunity to have a palliative care consultation and a palliative approach integrated into their care. We should also consider that the low number of patients being offered this consultation was partly due to the limited scope of the patient group, decided by the practitioners involved in the quality improvement initiative. It is also noteworthy that the interviewees highlighted several enabling factors, such as motivation and positive experiences, agreed routines and efficient solutions for communication. Relationship-building activities (e.g., being present) were also perceived as an enabling factor.

This study shows the importance of illuminating perceptions and beliefs (especially related to palliative care conceptualisation) in order to facilitate integration of a palliative consultation practice. A commonly discussed problem is the preconception that palliative care is equivalent to end-of-life care (Aldridge et al., 2016; Chosich et al., 2020; Friedrichsen et al., 2021; Kaasa et al., 2018), and this was reiterated in our study as a barrier to integration. Although there are examples of successful interventions for a change in such conceptual beliefs (Hahne et al., 2017), the present study’s findings imply that knowledge of the meaning of palliative care is not enough to overcome such barriers. It serves as a first step, but practical tools and measures to enhance context-specific communication skills are needed. Furthermore, since patient representatives also experienced that patients view ‘palliative’ as ‘end of life’, it seems crucial not only to address healthcare professionals’ perceptions, but also to make an effort to dispel myths regarding palliative care among patients and the public (Chosich et al., 2020).

There are multiple dimensions to the concept of integration about which the two teams appeared to both agree and disagree. The perception of *what* (Kaasa et al., 2018) should be integrated is related to the understanding of knowledge gaps within the organisation, as well as the understanding of the palliative care concept. Friedrichsen et al. (2021) have highlighted the divergence of views between a palliative care consultation team and healthcare professionals in acute care. Within out study, both the palliative care consultants, as well as a few surgical practitioners, believed that the surgeons’ intense focus on the surgical limited their knowledge and practice of palliative care. It is reasonable to assume that failing to identify palliative care needs could result in missed consultation opportunities. Hence, if surgeons (who lead the medical care) fail to identify the potential need for palliative care knowledge and/or the benefits of palliative care competence, integration between palliative care and surgical care may be hampered. Nevertheless, one surgeon in our study expressed appreciation for knowledge and skills stemming from collaboration with the palliative consultation team (mainly palliative care rounds), which indicates a knowledge translation of general palliative care.

Another important dimension of integration is *when* (Kaasa et al., 2018) something should be integrated, in this study illustrated by the perception of ‘*early’* integration, where the consultation team’s initial ambition of ‘early’ did not correspond with the surgical team’s expectations. This highlights the importance of clarifying the timing and concept of *‘early’* (Gaertner et al., 2011). However, the change in perception of the palliative care physician, acknowledging that it may be inappropriate to add another team when the patient was referred for oncological consultation, reflects the importance of contextual tailoring of early integration, avoiding a fixed definition of what is considered early, and what is considered integration of palliative care.

The palliative care consultation team saw several aspects of the palliative care and quality improvement practice as something that should be integrated in surgical practice, while the surgical team seemed to prefer a more autonomous consultation role, executing its own recommendations. For this reason, an important prerequisite for integration is consensus regarding the *how* (Kaasa et al., 2018), where perceptions regarding *who* is responsible for integration, *where* it is best integrated, and *how* it is best integrated need to come to light. An aggravating factor is that there seems to be no consensus within the palliative community about *how* and *when* to integrate (Hui & Bruera, 2015; Kaasa et al., 2018), or how the practices of palliative care consultation should be operationalised (Böling et al., 2020), which hampers the dissemination of palliative care.

Further, a shared view and understanding of roles between actors has been argued to be a prerequisite for successful collaboration (Firn et al., 2016; Gardiner et al., 2012; McDarby & Carpenter, 2019). Consistent with our results, Friedrichsen et al. (2021) also highlight actors as having differing expectations of the palliative care consultation team. Moreover, in line with findings in previous research (Böling et al., 2020), one of the perceived mission statements was for the palliative care consultant to have an educative role. This relates to a view of palliative care consultations as pro-active versus re-active, that is, is the consultation service here to ‘set up fire alarms or to extinguish full-scale fires’? Böling et al. (2020) concluded that palliative care consultation practice in Sweden largely seems to be a bottom-up driven practice due to premature national policy, possibly influencing differences in and misunderstandings about roles in practice.

This study highlights processes and influencing factors in the implementation of a palliative care consultation practice for a patient group that in general represents the traditionally assumed illness trajectory for people with cancer, with a short period of decline at the end-of-life. Nevertheless, the consultation role within this practice was perceived as not fully corresponding to patient needs. For example, the common rapid deterioration of patients in this group made this form of palliative care consultation appear to be misdirected, while a group of patients with a more stable and prolonged disease trajectory could perhaps have benefitted more from the quality improvement practice. This relates to the question of *how* and prompts questions regarding the limitations of the consultative role in relation to patient needs, and the need for disease-specific tailoring of palliative care consultations.

An important finding of our study – one that is rarely emphasised in the palliative care consultation literature – was the significance of the organisation of roles within each team in relation to the provision of palliative care, that is, *how* palliative care was carried out. Within the quality improvement initiative, the consultation model (as it was practised) seemed somewhat misdirected, encountering a different role distribution within the surgical team. Our findings also highlighted the central role of the clinical nurse specialist in the delivery of surgical palliative care. However, the quality improvement practices observed did not correspond to or fully leverage this role. Targeted interventions aimed at strengthening the palliative care competencies, conversation skills and formal mandate of clinical nurse specialists are essential for promoting the early integration of palliative care within surgical settings. These findings underscore the need to place greater emphasis on nursing expertise within palliative care consultation teams. Additionally, in relation to pro-activity, a more process- and change-oriented consultative approach, supporting palliative care processes within the patient-responsible care team, would likely benefit integration of palliative care.

Our findings further suggest that palliative care consultations cannot be the ‘only’ solution to palliative care provision; they need to be combined with a sufficiently developed specialised and non-specialised palliative care healthcare structure (Gaertner et al., 2012; Gaertner et al., 2011). This is in line with a public health palliative care perspective, which takes this thought further by suggesting an integration of specialised and non-specialised palliative care with civil society and informal networks (Abel et al., 2018). One suggestion could be to broaden the view of *who* palliative care consultants collaborate with. For example, closer collaboration with patient associations could enable not only better tailoring of interventions but also an opportunity to educate in palliative care and to present palliative care in a less stigmatised and dramatic way.

## Limitations

The initial ambition of this study was to gain a first-hand patient perspective by interviewing patients and recording patient consultations, but this was not possible due to the Covid pandemic. Instead, our patient perspective comes from patient association representatives who were not involved in the practice model, which prevents us from drawing conclusions regarding patient perspectives in relation to the specific quality improvement practice. Moreover, in terms of implementation of the palliative care practice, we have not assessed how and to what extent the practice was carried out, how many patients were available for inclusion, and the outcomes. This prevents us from drawing conclusions as to what works in clinical practice and the degree of integration of the practice. Further, given the extended duration of the quality improvement initiative and to gain a more comprehensive understanding of its development and implementation, it would have been beneficial to conduct interviews with healthcare professionals at multiple points throughout the project, rather than solely at its conclusion. The study would also have benefited from a larger number of healthcare professional interviewees. The quality improvement initiative also resided in a context where other collaborations between the consultation team and the surgical clinic preceded and were ongoing in parallel. This meant a challenge in the interview analysis to completely distinguish experiences of the specific quality improvement initiative from those stemming from a broader collaboration.

## Conclusion

This study illustrates how the interplay between surgical and palliative care teams, patient-family units, and organisational, societal, and implementation factors can shape both the development and success of a quality improvement project. Our findings suggest that perspectives on palliative care, views on integration and professional roles, as well as differences in palliative care practices and organisational structures, influenced the integration of palliative care within surgical cancer care through a consultation-based practice. Thus, within quality improvement initiatives aimed at integrating palliative care, expectations of *what*, *when* and *how* need to be explicitly addressed. Clear project structures and well-defined implementation measures are also essential to support such integration efforts. An early integrated palliative care health system organisation, especially for patients with pancreatic cancer, is dependent on both specialised and non-specialised interprofessional palliative care structures, tailored to the needs of the patients. Our study also underscored the significant role of the clinical nurse specialist in practising palliative care – a role that was insufficiently addressed in the quality improvement initiative – highlighting the need to align palliative care consultation practices with the organisational structures and specific needs of the collaborating team. Behind the barriers disclosed is a need for a supportive policy for palliative consultation services that includes how to contextually adapt and tailor them. Future palliative care consultation initiatives and research should consider a process-oriented approach where perceptions, care practice and organisational aspects relevant to palliative care are considered.

## Supplemental Material

sj-pdf-1-gqn-10.1177_23333936261421581 – Supplemental material for Early Integration of Palliative Care in Hospitals: How Can Palliative Care Consultation Teams Drive Practice Change? Supplemental material, sj-pdf-1-gqn-10.1177_23333936261421581 for Early Integration of Palliative Care in Hospitals: How Can Palliative Care Consultation Teams Drive Practice Change? by Susanna Böling, My Engström, Johan Berlin and Joakim Öhlén in Global Qualitative Nursing Research

sj-pdf-2-gqn-10.1177_23333936261421581 – Supplemental material for Early Integration of Palliative Care in Hospitals: How Can Palliative Care Consultation Teams Drive Practice Change? Supplemental material, sj-pdf-2-gqn-10.1177_23333936261421581 for Early Integration of Palliative Care in Hospitals: How Can Palliative Care Consultation Teams Drive Practice Change? by Susanna Böling, My Engström, Johan Berlin and Joakim Öhlén in Global Qualitative Nursing Research

sj-pdf-3-gqn-10.1177_23333936261421581 – Supplemental material for Early Integration of Palliative Care in Hospitals: How Can Palliative Care Consultation Teams Drive Practice Change? Supplemental material, sj-pdf-3-gqn-10.1177_23333936261421581 for Early Integration of Palliative Care in Hospitals: How Can Palliative Care Consultation Teams Drive Practice Change? by Susanna Böling, My Engström, Johan Berlin and Joakim Öhlén in Global Qualitative Nursing Research
