# Supplementary material for: Early Integration of Palliative Care in Hospitals: How Can Palliative Care Consultation Teams Drive Practice Change?
Source: Glob Qual Nurs Res. 2026 Feb 20;13:23333936261421581. doi: 10.1177/23333936261421581 (PMC12925021; doi:10.1177/23333936261421581)
Supplement: sj-pdf-1-gqn-10.1177_23333936261421581 – Supplemental material for Early Integration of Palliative Care in Hospitals: How Can Palliative Care Consultation Teams Drive Practice Change? [file sj-pdf-1-gqn-10.1177_23333936261421581.pdf]

Supplementary file 1

The quality improvement initiative

A practice-led, quality improvement initiative was initiated in May 2019 for patients with non-curable pancreatic cancer in a larger city in Sweden. The patients were cared for in a specialised surgical clinic (one out-patient unit and one in-patient ward) in a university hospital, with approximately 1,600 beds. This was a joint project between a palliative care consultation service and the surgical clinic, and seen as an opportunity to develop an already existing collaboration comprising regular palliative care consultation rounds in the surgical in-patient ward. For the palliative care consultation team, the quality improvement was a means of developing strategies to apply early integrated palliative care within the hospital, while the surgical team perceived a need to change care practice for patients with pancreatic cancer, who were a vulnerable group and often fell between the cracks and received specialised palliative care to late, if at all. The aim of the quality improvement was to design and test a model for early integrated palliative care through palliative care consultations. In this project, 'early' meant close to when the patient received information about their diagnosis. The teams jointly decided the target patient group to comprise adult patients with pancreatic cancer who at diagnosis were considered as neither benefiting from surgery nor chemotherapy. At a later stage of the project, this group was deemed too small, with an insufficient number of available patients, and was therefore expanded to also include patients who at diagnosis were considered for oncological treatment and referred to the oncological department, but for whom the oncologist judged chemotherapy to be of no benefit.

A cyclical approach was applied to developing, testing and evaluating the quality improvement practice (Coghlan & Brannick, 2014). During the course of the quality improvement, meetings were repeatedly held between the surgical team and the palliative care consultation team with the intention of evaluating the collaboration and quality improvement practice and responding and adapting to identified barriers and needs. Initially, much of the discussion focused on how to increase the number of included patients. After expanding the target patient group, more patients were offered a palliative care consultation and the discussions focused more on the collaboration itself. During the course of the quality improvement, changes mainly concerned which specific patient group to include, a switch to digital patient consultations (due to the Covid pandemic), ways of engaging the surgical in-patient ward in the quality improvement, internal routines, and communication. Healthcare professionals in the surgical clinic were informed about the new quality improvement practice prior to its launch in February 2020. Shortly after its introduction, the quality

improvement paused due to the Covid pandemic (March 2020), commencing again in October 2020 and continuing until April 2022. During the course of the project, the palliative care consultation team saw around 14 patients, including men and women aged between 70-95 (approx. 3 missing).

The research team followed the quality improvement initiative from its developmental stage to the end and participated in the initial discussions, the launch and follow-up of the project. The researchers shared the coordinating role with the consultation team during the development phase of the quality improvement. They held informal check-ins with individual members of both teams, particularly during the early implementation phase, and were involved in organising the follow-up meetings. However, responsibility for the quality-improvement project lay jointly with the palliative care consultation team and the surgical clinic, who led the development and integration of the practice. One of the co-authors of this paper was affiliated with the surgical clinic, and another affiliated with the palliative care centre, from which the consultation team operated.

Quality-improvement practice design

Within the surgical clinic, care practice for patients with advanced pancreatic cancer had previously involved an initial outpatient visit in which a surgeon and a clinical nurse specialist[1] informed the patient about their diagnosis and level of treatment (explicitly labelled 'best supportive care'). There were no routine follow-ups, but the patient was encouraged to contact the clinical nurse specialist in the outpatient clinic when needed. If required during their illness trajectory, the patient would receive surgical in-patient care, referral to domiciliary nursing care or referral for hand over to specialised palliative care. There was also an opportunity for the patient to visit the surgical day-clinic or book a physician consultation.

Within the quality improvement change, the clinical nurse specialist offered patients a palliative care consultation as a complement to usual care practice. The offer was primarily presented during the outpatient consultation when the diagnosis was communicated. The palliative care consultation team comprised a physician (usually with palliative medicine specialisation or extensive palliative care experience), a registered nurse (usually with specialist education), and a social worker. The consultation itself was tailored to the patient's needs, and included an assessment of symptoms, appropriate level of care and information regarding possible supportive healthcare, societal and community resources (Table 1). The interprofessional palliative care consultation team had a consultative role with no patient responsibility and offered their recommendations for

[1] Known as "contact nurse" in Swedish, which is the patient's primary healthcare contact (Sharp et al., 2018). A function that follows Swedish national healthcare policy (Regionala cancercentrum i samverkan [Regional Cancer Centres in Sweden], 2021; Socialdepartementet [Ministry of Social Affairs], 2009).

implementation by the surgical team responsible for the patient's care. Recommendations were documented in the patient record and communicated to the clinical nurse specialist. If needed, the clinical nurse specialist contacted other healthcare professionals (e.g. a surgeon) to implement recommendations such as medical treatment, certificate for family carer allowance, or referrals to specialised palliative care or home care services. The palliative care consultation team was then available for further consultation with the surgical clinic, if required.

Presentation – *social worker*<br>1. Social worker<br>2. Nurse<br>3. Physician	• What is the idea behind this consultation?<br>• We have read your patient record<br>• We have set aside around 45 min<br>• We offer an advisory service, not treatment<br>• What expectations do you have of our meeting today?
The current situation – *nurse*<br><br>**ESAS** (Edmonton Symptom Assessment Scale)<br><br>*physician*	• How are you feeling today?<br>• Any changes?<br>• What are your thoughts about the future?<br>• Would you like more information?<br><br>• Symptom relief, medication, and more.<br><br>*Refer back to the out-patient surgery clinic*
What is there to offer? – *nurse*<br><br>*social worker*	• Domiciliary care /personal alarms<br>• Domiciliary nursing care<br>• Specialised palliative home care<br>• Short term residential care home<br>• Hospice – awaiting referral<br>• Hospital readmission<br><br>• Special transportation services for the disabled<br>• Family carer allowance<br>• Counselling contact with social worker?
Summary – *physician*	• Would you like us to send you more information?<br>• Anything else?

Table 1. Consultation outline. Developed by the palliative care consultation team.

References

Coghlan, D., & Brannick, T. (2014). *Doing action research in your own organization (4th ed.)*. SAGE.

Regionala cancercentrum i samverkan [Regional Cancer Centres in Sweden]. (2021). Bukspottkörtelcancer - Nationellt vårdprogram 3.1 [Pancreatic Cancer - National Clinical

Practice Guidelines 3.1]. https://kunskapsbanken.cancercentrum.se/globalassets/cancerdiagnoser/bukspottkortel/vardprogram/nvp-bukspottkortelcancer.pdf

Sharp, L., Westman, B., Olofsson, A., Leppänen, A., & Henriksson, R. (2018). Access to supportive care during and after cancer treatment and the impact of socioeconomic factors. *Acta oncologica, 57*(10), 1303-1310. https://doi.org/10.1080/0284186X.2018.1484157

Socialdepartementet [Ministry of Social Affairs]. (2009). *Statens offentliga utredningar - En nationell cancerstrategi för framtiden (SOU 2009:11) [Swedish Government Official Reports - A National Cancer Strategy for the Future (SOU 2009:11)]*. https://www.regeringen.se/rattsliga-dokument/statens-offentliga-utredningar/2009/02/sou-200911/
