# Supplementary material for: Early Integration of Palliative Care in Hospitals: How Can Palliative Care Consultation Teams Drive Practice Change?
Source: Glob Qual Nurs Res. 2026 Feb 20;13:23333936261421581. doi: 10.1177/23333936261421581 (PMC12925021; doi:10.1177/23333936261421581)
Supplement: sj-pdf-2-gqn-10.1177_23333936261421581 – Supplemental material for Early Integration of Palliative Care in Hospitals: How Can Palliative Care Consultation Teams Drive Practice Change? [file sj-pdf-2-gqn-10.1177_23333936261421581.pdf]

Supplementary file 2 – Interview guide

Patient responsible team/palliative care consultation team:

- Could you tell me how the implementation of the practice turned out?
- Could you tell me what factors led to the result?
- Could you tell me what you have learned from this approach that you would like to apply to future strategies for this patient group? (If you were to redo or start a new project, how would it be structured to meet the needs of your organisation and patients with pancreatic cancer)/ Please tell us what you have learned from this approach that you would like to apply to future consultation practices.
- Could you tell me if there is any other aspect that you find relevant that we haven't discussed during the interview?

Managers surgical department/manager palliative care consultation team:

- Could you tell me how the palliative care in your organisation/at the hospital works today?
- What factors influence palliative care in your organisation/at the hospital?
- Please tell me about any changes you would like to see in relation to (early integration of) palliative care. (Surgical department only)
- What role can palliative care consultants play in the integration of palliative care at the hospital?

Patient association representatives

- What are your perceptions of the need for support among people with pancreatic cancer and their family, particularly during the transition from curative to palliative care and during the palliative phase?

- What is your view on how the care continuum and the collaboration between different healthcare providers and community actors work? (What works, what doesn't?)
- As we know, pancreatic cancer is a serious illness, often with a grim prognosis. Please share your thoughts on the transition from curative to palliative care, where the focus shifts to well-being and quality of life.
- Returning to the approach of using palliative care consultants, which has been tested in the clinic, what are your initial thoughts on such an approach from your perspective?

Healthcare professionals at the in-patient ward

- Could you tell me how palliative care in the ward/clinic works today?
- What factors influence palliative care at the clinic?
- Please tell me about any changes you would like to see in relation to (early integration of) palliative care.
- Could you tell me how the implementation of the practice with palliative care consultations for patients with pancreatic cancer turned out?
