# Supplementary material for: Early Integration of Palliative Care in Hospitals: How Can Palliative Care Consultation Teams Drive Practice Change?
Source: Glob Qual Nurs Res. 2026 Feb 20;13:23333936261421581. doi: 10.1177/23333936261421581 (PMC12925021; doi:10.1177/23333936261421581)
Supplement: sj-pdf-3-gqn-10.1177_23333936261421581 – Supplemental material for Early Integration of Palliative Care in Hospitals: How Can Palliative Care Consultation Teams Drive Practice Change? [file sj-pdf-3-gqn-10.1177_23333936261421581.pdf]

Supplementary file 3

Perceived barriers and enablers influencing the processes, collaboration and impact of the palliative care consultation practice for early integrated palliative care

	Barriers	Enablers
Prerequisites	• Lack of clarity and structure within the quality improvement project<br>• Lack of information and communication<br>• Lack of knowledge about the project and too much reliance on significant individuals<br>• Lack of committed nurses within the in-patient ward, and surgeons in general<br>• Lack of motivation within the consultation team related to a perception of the project being inert, a resource demanding practice, and greater costs than gains<br>• Time constraints	• Establishment of administrative routines<br>• Motivation within the surgical team and seeing the integrated consultation practice as something positive and beneficial for the patient.<br>• Consultant advice relevant to the surgical practice<br>• Clear information regarding the project at the outset<br>• Perceived need for support regarding palliative care issues within the surgical clinic
Process of offering the opportunity to patients	• Remembering the project in a strained work environment<br>• Challenges in asking the patients due to healthcare professionals' own perceptions of palliative care and the "right time" to ask them	• Sometimes perceived as a natural thing to offer<br>• Internal and external facilitators within the clinical nurse specialist group that reminded them about the project

	• Lack of supporting tools, such as pamphlets to hand out to the patient to read when it suited them.<br>• Lack of training and support regarding palliative care conversations with patients<br>• No routines for patient follow up = missed opportunities<br>• Limited suitable patient group to ask<br>• Uncertainty regarding what patient group to include<br>• The patient had other needs or had to be referred for hand over to specialised palliative care<br>• Not always clear if the patient knew their treatment intent was palliative (in-patient ward)	• Motivation, positive feelings towards the project practice and viewing it as a possibility when the patient lived in an area without access to specialised palliative homecare.<br>• Better chance of remembering when a clinical nurse specialist participated in the surgical consultation with the patient<br>• Broadening of the patient group<br>• A numeric target of patients to be invited
Attributes of the patients' disease	• Limited time to meet the patients due to severe illness and fast deterioration<br>• The quality improvement practice was not well suited to the most severely ill<br>• One factor too many for a severely ill patient	
Collaboration between the surgical clinic and the palliative consultation team	• Insufficient communication regarding patient recommendations<br>• Divergent perspectives on patient needs and palliative care practices	• Establishment of routines<br>• Digital communication tools<br>• Communication<br>• Establishment of a relationship between the actors<br>• Meetings in person between teams

	• The palliative care consultation team not implementing their own recommendations<br>• Lack of communication with the surgical in-patient ward regarding the project<br>• Lack of physical presence and relationship building activities<br>• The quality improvement practice being too formal	• A designated time for palliative consultations in the surgical clinic<br>• Important advice from the palliative care consultation team communicated from the clinical nurse specialist to the responsible surgeon
Process of palliative care consultation with patients	• Changes in the external organisation of specialised palliative care that obstructed the consultation team's knowledge of supportive services for the patient and family	• Establishment of a patient conversation outline<br>• Digital tools for patient consultation during the Covid pandemic enabled communication and made consultations more time efficient<br>• Being a team, and familiarity within the consultation team<br>• A flexible consultation team (implementing recommendations) could result in better patient outcomes
Contextual factors	• Covid<br>- Fewer patients<br>- The project paused before it was established<br>- Priorities other than the quality improvement project and constant changes in routines<br>- Information fatigue<br>- Too wide a distance between the palliative care consultants and the surgical clinic	• Support from managers

	- Change of workplace for healthcare staff<br>- New routines within the quality improvement project<br>• Hospital work environment and healthcare professional turn over<br>• Other parallel changes<br>• Organisational changes in specialised palliative care in the community	
